# Identification of hepatoblastoma susceptibility loci in the *TRMT6* gene from a seven‐center case–control study

**DOI:** 10.1111/jcmm.18006

**Published:** 2023-10-18

**Authors:** Lin Ma, Jinhong Zhu, Jiao Zhang, Wenli Zhang, Yong Li, Zhonghua Yang, Suhong Li, Jiwen Cheng, Li Li, Jing He, Peng Liu

**Affiliations:** ^1^ Department of Clinical Laboratory The First Affiliated Hospital of Zhengzhou University, Key Clinical Laboratory of Henan Province Zhengzhou Henan China; ^2^ Department of Clinical Laboratory Biobank, Harbin Medical University Cancer Hospital Harbin Heilongjiang China; ^3^ Department of Pediatric Surgery the First Affiliated Hospital of Zhengzhou University Zhengzhou Henan China; ^4^ Department of Pediatric Surgery Guangzhou Institute of Pediatrics, Guangdong Provincial Key Laboratory of Research in Structural Birth Defect Disease, Guangzhou Women and Children's Medical Center, Guangzhou Medical University Guangzhou Guangdong China; ^5^ Department of Pediatric Surgery Hunan Children's Hospital Changsha Hunan China; ^6^ Department of Pediatric Surgery Shengjing Hospital of China Medical University Shenyang Liaoning China; ^7^ Department of Pathology Children Hospital and Women Health Center of Shanxi Taiyuan Shannxi China; ^8^ Department of Pediatric Surgery the Second Affiliated Hospital of Xi'an Jiaotong University Xi'an Shaanxi China; ^9^ Kunming Key Laboratory of Children Infection and Immunity, Yunnan Key Laboratory of Children's Major Disease Research Yunnan Institute of Pediatrics Research, Yunnan Medical Center for Pediatric Diseases, Kunming Children's Hospital Kunming Yunnan China; ^10^ Department of Pediatric Intensive Care Unit the First Affiliated Hospital of Zhengzhou University Zhengzhou Henan China

**Keywords:** hepatoblastoma, m^1^A modification, polymorphism, susceptibility, *TRMT6*

## Abstract

Hepatoblastoma, the most frequently diagnosed primary paediatric liver tumour, bears the lowest somatic mutation burden among paediatric neoplasms. Therefore, it is essential to identify pathogenic germline genetic variants, especially those in oncogenic genes, for this disease. The tRNA methyltransferase 6 noncatalytic subunit (TRMT6) forms a tRNA methyltransferase complex with TRMT61A to catalyse adenosine methylation at position N1 of RNAs. TRMT6 has displayed tumour‐promoting functions in several cancer types. However, the contribution of its genetic variants to hepatoblastoma remains unclear. In this study, we investigated the association between four *TRMT6* polymorphisms (rs236170 A > G, rs451571 T > C, rs236188 G > A and rs236110 C > A) and the risk of hepatoblastoma in a cohort of 313 cases and 1446 healthy controls. Germline DNA was subjected to polymorphism genotyping via the TaqMan qPCR method. Odds ratio (OR) and 95% confidence interval (CI) were used to determine hepatoblastoma susceptibility variants. The rs236170 A > G, rs236188 G > A and rs236110 C > A polymorphisms were significantly associated with hepatoblastoma risk. Combination analysis of the four polymorphisms revealed that children bearing 1–4 risk genotypes were at significantly enhanced hepatoblastoma risk compared to those without risk genotype (adjusted OR = 1.52, 95% CI = 1.19–1.95, *p* = 0.0008). We also conducted stratification analyses by age, sex and clinical stage. Ultimately, we found that the rs236110 C > A was significantly associated with the downregulation of MCM8, a neighbouring gene of *TRMT6*. In conclusion, we identified three susceptibility loci in the *TRMT6* gene for hepatoblastoma. Our findings warrant further validation by extensive case–control studies across different ethnicities.

## 1 INTRODUCTION

N1‐methyladenosine (m^1^A), one of the chemical modifications, is located on the first nitrogen atom of adenosine in RNA. m^1^A is present ubiquitously in mRNA, rRNA, lncRNA and tRNA but is most enriched in tRNA. By affecting RNA base pairing, m^1^A profoundly impacts RNA's structure, stability, translation and function. For instance, m^1^A at position 58 of tRNA is critical for maintaining the proper structure of tRNA and stability and starting the translational process; lack of m^1^A modification in this site was reported to induce tRNA‐derived small RNAs (tDRs), facilitating ribosome assembly and leading to malignant transformations.^1^ Several methyltransferases that catalyse RNA m^1^A modification have been identified, including the tRNA methyltransferase 6 (TRMT6), TRMT61A, TRMT61B, TRMT10C and NML.^2^ TRMT6 and TRMT61A form a functional heterotetramer complex to deposit N^1^‐methylation in target RNA. TRMT6, the noncatalytic subunit of the methyltransferase complex, is responsible for tRNA binding, while TRMT61A, carrying a methyl donor binding pouch, acts as the catalytic subunit.^2^ Recently, increasing evidence indicates that TRMT6 is preferentially expressed in cancerous tissues and plays an oncogenic role in various types of cancer, such as glioma,^3^ bladder cancer^1^ and hepatocellular carcinoma (HCC).^4^, ^5^

Hepatoblastoma, the most common primary paediatric liver malignancy, is extremely rare, with an annual incidence varying from 1.2 to 1.5 cases per million.^6^ In particular, it was estimated that the incidence rate of hepatoblastoma is about 1.4 per million Chinese children yearly.^7^ Hepatoblastoma is an embryonal tumour arising from hepatoblasts, which often exhibits mixed histological patterns representing different developmental stages of the liver. Several factors seem to increase the risk of hepatoblastoma, including inferior birth status (premature birth and very low birth), some treatments (Oxygen therapy, furosemide, total parenteral nutrition [TPN] and radiation) and toxins (e.g. plasticizers). Besides, hereditary predispositions also contribute to the development of hepatoblastoma. For instance, several constitutional genetic syndromes have shown associations with increased hepatoblastoma risk, such as Trisomy 18/Edward's syndrome, Beckwith–Wiedemann syndrome (BWS) and familial adenomatous polyposis (FAP). Unlike adult tumours with high somatic mutation prevalence, germline variants in cancer susceptibility genes are often reported in paediatric cancers, which may contribute to 8%–10% of paediatric tumours. Additionally, previous findings indicate that hepatoblastoma harbours the fewest somatic mutations out of all solid childhood tumours, underlying the importance of genetic variants in the pathogenesis of hepatoblastoma.^8^ Other and our research teams have identified hepatoblastoma susceptibility genetic variants in many genes, including *MPO*, *CCND1*, *LIN28B*, *HMGA2*, *XPC*, *YTHDF1*, *YTHDC1*, *WTAP*, *WDR4* and *METTL1*.^9^, ^10^, ^11^, ^12^, ^13^, ^14^, ^15^, ^16^, ^17^, ^18^

Although different research teams confirmed the contributing role of TRMT6 in the carcinogenesis of HCC,^4^, ^5^ its impacts on hepatoblastoma are unknown. Besides, no studies have reported the associations between genetic polymorphisms of the *TRMT6* gene and the risk of hepatoblastoma. Our research aims to identify pathogenic genetic polymorphisms for hepatoblastoma in Chinese children with a cohort of 313 cases and 1446 healthy controls.

## 2 MATERIALS AND METHODS

### 2.1 Patient and study design

Patients were diagnosed with hepatoblastoma as manifested by evidence from clinical examinations, laboratory testing, pathological examination and imaging. Sufficient peripheral whole blood samples were obtained from participants for analysis. Cases (*n* = 313) were all Han Chinese descendants younger than 14 years of age. They were diagnosed in seven independent hospitals in Guangzhou, Kunming, Changsha, Taiyuan, Xi'an, Zhengzhou and Shenyang. Children who underwent health check‐ups in those hospitals during a similar period were recruited as healthy controls (*n* = 1446) to minimize selection bias. Patients and controls were matched concerning age and sex (Table S1).^12^ We staged the patients according to the PRETEXT classification.^19^ All patients offered informed consent for molecular research before being recruited. The study was conducted with a protocol (No: 202016601) authorized by the institutional review board of Guangzhou Women and Children's Medical Center. Participants' epidemiological and clinical characteristics were described previously.^12^

### 2.2 Genotyping and selecting polymorphisms

We arbitrarily chose the candidate single nucleotide polymorphisms (SNPs) for this study from the dbSNP database (http://www.ncbi.nlm.nih.gov/projects/SNP) following the previously published criteria.^20^, ^21^, ^22^ Only SNPs having potential biological functions, as suggested by SNPinfo (https://snpinfo.niehs.nih.gov), were qualified for the study. Moreover, we only chose SNPs with low linkage disequilibrium (LD) (*R* ^2^ < 0.8) (https://ldlink.nih.gov/?tab=ldmatrix). The four *TRMT6* polymorphisms (rs236170 A > G, rs451571 T > C, rs236188 G > A and rs236110 C > A) showed low LD with each other, with *R* ^2^ varying from 0.066 to 0.732. Regarding potential biological functions, the rs236110 C > A is located in an exonic splicing enhancer or exonic splicing silencer, to which a specialized protein binds to elevate or decrease the efficiency of exon inclusion. The rs236170 A > G in the miRNA binding site of the *TRMT6* gene may impact the stability of its transcripts. The rs236188 G > A in the transcription factor binding site may potentially alter the affinity between certain transcription factors and the promoters of the *TRMT6* gene. Finally, the rs451571 T > C and rs236110 C > A are missense variants in the coding sequence and may lead to changes in amino acids during translation. Genomic DNA was extracted from participants' peripheral blood samples donated before treatment using the Tiangen Blood DNA Extraction kits (Tiangen Biotechnology). Genotyping assays were performed using Taqman qPCR on a TaqMan platform (Applied Biosystems).

### 2.3 Statistical analysis

We first performed a goodness‐of‐fit chi‐square test to check these SNPs' Hardy–Weinberg equilibrium (HWE) in the controls. Next, we assessed the SNPs' associations with hepatoblastoma susceptibility using the logistic regression analysis after adjustment for age, sex and clinical stage. The resulting odds ratio (OR) and 95% confidence interval (CI) were used to evaluate the significance of the associations. In the multivariate analyses, age, sex and the clinical stage were used as adjusted covariates. The following genetic models were employed to evaluate the association between the four SNPs and hepatoblastoma susceptibility: homozygous (WW vs. VV), heterozygous (WW vs. WV), dominant (WW vs. WV/VV) and recessive (WW/WV vs. VV) models. W and V depicted wild type and variant alleles of an SNP, respectively. The stratified analyses by age, sex and clinical stage were also carried out. In the last, we investigated the association between the above SNPs and expression levels of relevant genes, that is, expression quantitative trait locus (eQTL), using a web tool based on Genotype‐Tissue Expression (GTEx) project.^23^ All analyses were two‐sided using SAS v9.1 software (SAS Institute Inc., Cary, NC), and a significance level of 0.05 was adopted.

## 3 RESULTS

### 3.1 Association study

Overall, 310 cases and 1444 healthy controls were genotyped successfully among the 313 cases and 1446 controls. Four potential functional *TRMT6* polymorphisms (rs236170 A > G, rs451571 T > C, rs236188 G > A and rs236110 C > A) were successfully genotyped and analysed for their contributions in hepatoblastoma susceptibility. The results are summarized in Table 1. We first performed a single locus analysis after confirming that the genotype distributions of these SNPs were not divergent from the Hardy–Weinberg equilibrium. Multivariate regression analysis demonstrated that three *TRMT6* polymorphisms (rs236170 A > G, rs236188 G > A and rs236110 C > A) were significantly associated with susceptibility to hepatoblastoma (Table 1). Significant associations with decreased hepatoblastoma risk were observed under the heterozygous (adjusted OR = 0.74, 95% CI = 0.56–0.97, *p* = 0.031) and dominant (adjusted OR = 0.77, 95% CI = 0.59–0.99, *p* = 0.039) models for rs236170 A > G, but under the heterozygous (adjusted OR = 0.68, 95% CI = 0.47–0.99, *p* = 0.043) model only for rs236188 G > A. Intriguingly, rs236110 C > A showed either protective or detrimental effects under different genetic models, including the heterozygous (adjusted OR = 0.75, 95% CI = 0.57–0.997, *p* = 0.048), homozygous (adjusted OR = 2.76, 95% CI = 1.65–4.61, *p* = 0.0001) and recessive (adjusted OR = 3.01, 95% CI = 1.81–5.00, *p* < 0.0001) models. Moreover, we defined rs236170 AA, rs451571 CC, rs236188 AA and rs236110 AA as risk genotypes. The integrative analyses indicated that 1–4 risk genotypes significantly conferred hepatoblastoma susceptibility (adjusted OR = 1.52, 95% CI = 1.19–1.95, *p* = 0.0008).

**TABLE 1:** Association of *TRMT6* gene polymorphisms with hepatoblastoma susceptibility.

Genotype	Cases (*N* = 310)	Controls (*N* = 1444)	*P* ^a^	Crude OR (95% CI)	*P*	Adjusted OR (95% CI^b^	*P* ^b^
rs236170 A > G (HWE = 0.012)							
AA	121 (39.03)	475 (32.89)		1.00		1.00	
AG	125 (40.32)	665 (46.05)		**0.74 (0.56–0.97)**	**0.031**	**0.74 (0.56–0.97)**	**0.031**
GG	64 (20.65)	304 (21.05)		0.83 (0.59–1.16)	0.265	0.83 (0.59–1.16)	0.269
Additive			0.152	0.88 (0.75–1.05)	0.152	0.88 (0.75–1.05)	0.154
Dominant	189 (60.97)	969 (67.11)	0.038	**0.77 (0.59–0.99)**	**0.039**	**0.77 (0.59–0.99)**	**0.039**
Recessive	246 (79.35)	1140 (78.95)	0.873	0.98 (0.72–1.32)	0.874	0.98 (0.72–1.32)	0.884
rs451571 T > C (HWE = 0.118)							
TT	198 (63.87)	871 (60.32)		1.00		1.00	
TC	92 (29.68)	514 (35.60)		0.79 (0.60–1.03)	0.083	0.79 (0.60–1.04)	0.090
CC	20 (6.45)	59 (4.09)		1.49 (0.88–2.53)	0.140	1.48 (0.87–2.51)	0.149
Additive			0.744	0.97 (0.78–1.19)	0.744	0.97 (0.78–1.20)	0.753
Dominant	112 (36.13)	573 (39.68)	0.245	0.86 (0.67–1.11)	0.245	0.86 (0.67–1.11)	0.258
Recessive	290 (93.55)	1385 (95.91)	0.068	1.62 (0.96–2.73)	0.071	1.60 (0.95–2.70)	0.079
rs236188 G > A (HWE = 0.477)							
GG	270 (87.10)	1199 (83.03)		1.00		1.00	
GA	36 (11.61)	236 (16.34)		**0.68 (0.47–0.99)**	**0.042**	**0.68 (0.47–0.99)**	**0.043**
AA	4 (1.29)	9 (0.62)		1.97 (0.60–6.46)	0.261	1.91 (0.58–6.25)	0.287
Additive			0.169	0.79 (0.57–1.11)	0.170	0.79 (0.57–1.10)	0.169
Dominant	40 (12.90)	245 (16.97)	0.079	0.73 (0.51–1.04)	0.080	0.73 (0.51–1.04)	0.080
Recessive	306 (98.71)	1435 (99.38)	0.214	2.08 (0.64–6.81)	0.224	2.01 (0.61–6.59)	0.249
rs236110 C > A (HWE = 0.036)							
CC	205 (66.13)	925 (64.06)		1.00		1.00	
CA	79 (25.48)	477 (33.03)		**0.75 (0.56–0.99)**	**0.043**	**0.75 (0.57–0.997)**	**0.048**
AA	26 (8.39)	42 (2.91)		**2.79 (1.67–4.66)**	**<0.0001**	**2.76 (1.65–4.61)**	**0.0001**
Additive			0.333	1.11 (0.90–1.38)	0.333	1.11 (0.90–1.38)	0.325
Dominant	105 (33.87)	519 (35.94)	0.490	0.91 (0.71–1.18)	0.490	0.92 (0.71–1.19)	0.518
Recessive	284 (91.61)	1402 (97.09)	<0.0001	**3.06 (1.84–5.07)**	**<0.0001**	**3.01 (1.81–5.00)**	**<0.0001**
Risk genotypes^c^							
0	163 (52.58)	908 (62.88)		1.00		1.00	
1–4	147 (47.42)	536 (37.12)	0.0007	**1.53 (1.19–1.96)**	**0.0008**	**1.52 (1.19–1.95)**	**0.0008**

*Note:* Values were in bold if the *P* 〈 0.05 or the 95 % CI excluding 1.00.

Abbreviations: CI, confidence interval; HWE, Hardy–Weinberg equilibrium; OR, odds ratio.

^a^ Chi‐square test for genotype distributions between hepatoblastoma patients and cancer‐free controls.

^b^ Adjusted for age and sex.

^c^ Risk genotypes were rs236170 AA, rs451571 CC, rs236188 AA and rs236110 AA.

### 3.2 Stratification analysis

We also stratified the association study by age, sex and clinical stage (Table 2). Under the dominant genetic model, the association between rs236170 and hepatoblastoma susceptibility remained significant in the subgroup of I + II stages (adjusted OR = 0.66, 95% CI = 0.48–0.92, *p* = 0.015). Regarding the recessive genetic model, the rs236110 was significantly associated with the risk of hepatoblastoma, regardless of age, sex and clinical stage. Finally, 1–4 risk genotypes significantly contributed to hepatoblastoma predisposition among both age groups, boys and subgroups of clinical I + II stages (Table 2).

**TABLE 2:** Stratification analysis of the association between *TRMT6* genotypes and hepatoblastoma susceptibility.

Variables	rs236170 (cases/controls)		AOR (95% CI)^a^	*P* ^a^	rs236110 (cases/controls)		AOR (95% CI)^a^	*P* ^a^	Risk genotypes (cases/controls)		AOR (95% CI)^a^	*P* ^a^
	AA	AG/GG			CC/CA	AA			0	1–4		
Age, month												
<17	64/206	103/435	0.77 (0.54–1.09)	0.140	152/620	15/21	**2.96 (1.49–5.88)**	**0.002**	87/404	80/237	**1.56 (1.11–2.20)**	**0.011**
≥17	57/269	86/534	0.76 (0.53–1.10)	0.141	132/782	11/21	**3.15 (1.48–6.69)**	**0.003**	76/504	67/299	**1.49 (1.04–2.13)**	**0.030**
Sex												
Females	49/191	79/404	0.76 (0.51–1.13)	0.176	118/577	10/18	**2.72 (1.22–6.07)**	**0.015**	71/374	57/221	1.36 (0.92–2.00)	0.119
Males	72/284	110/565	0.77 (0.56–1.07)	0.124	166/825	16/24	**3.26 (1.70–6.28)**	**0.0004**	92/534	90/315	**1.65 (1.19–2.27)**	**0.003**
Clinical stages												
I + II	68/475	92/969	**0.66 (0.48–0.92)**	**0.015**	144/1402	16/42	**3.65 (2.00–6.66)**	**<0.0001**	79/908	81/536	**1.73 (1.25–2.41)**	**0.001**
III + IV	33/475	58/969	0.86 (0.56–1.34)	0.512	83/1402	8/42	**3.25 (1.47–7.16)**	**0.004**	48/908	43/536	1.52 (0.99–2.33)	0.054

*Note:* Values were in bold if the *P* 〈 0.05 or the 95 % CI excluding 1.00.

Abbreviations: AOR, adjusted odds ratio; CI, confidence interval.

^a^ Adjusted for age and sex, omitting the corresponding stratification factor.

### 3.3 Expression quantitative trait loci (eQTL) analysis

**FIGURE 1:**
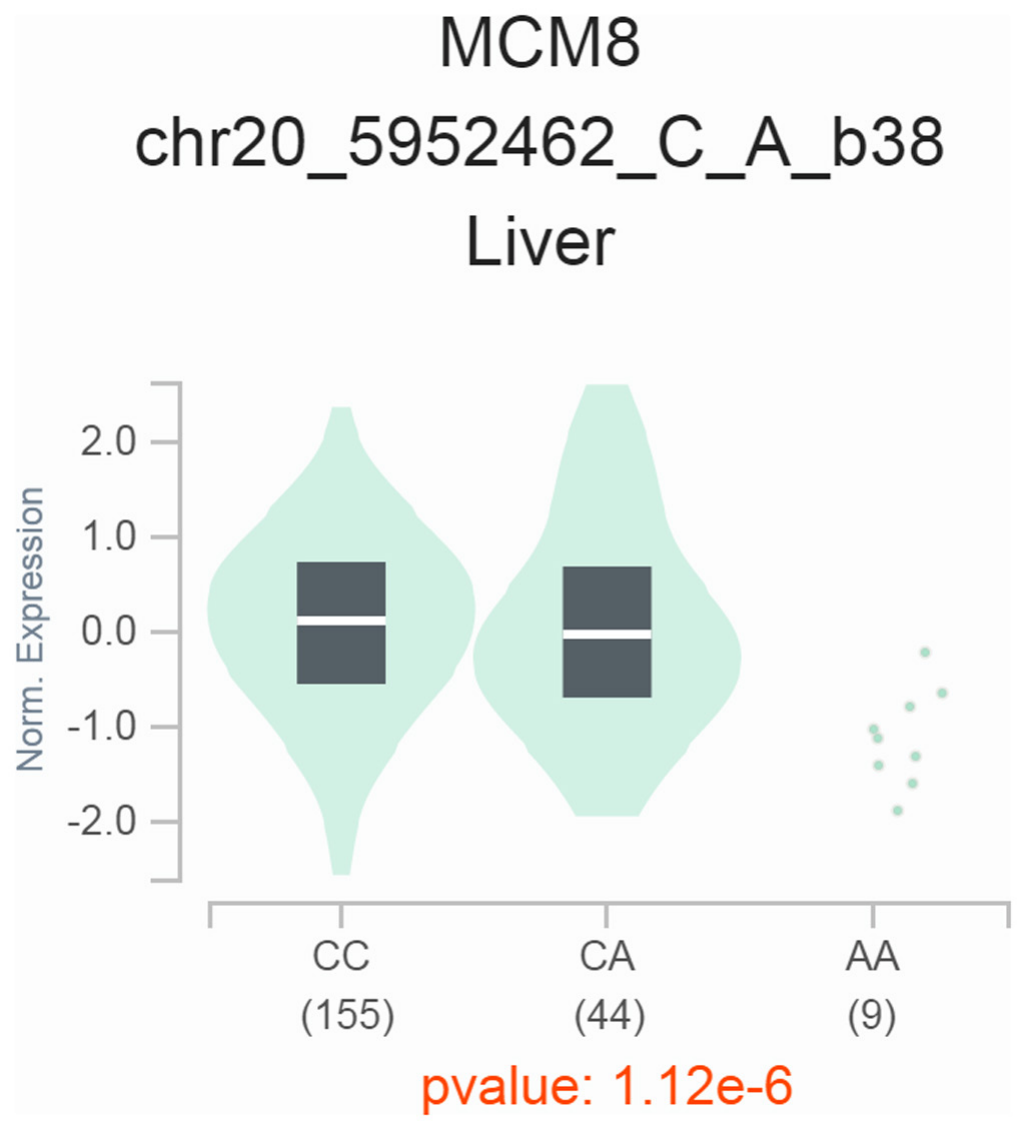
GTEx analysis for the association between *TRMT6* rs236110 C > A polymorphism and *MCM8* gene expression in liver tissue.

We finally interrogated whether the significant SNPs affected the expression of the *TRMT6* gene or its nearby genes. The data from the GTEx website unveiled that the rs236110 C > A polymorphism was related to the altered expression of the *minichromosome maintenance 8* (*MCM8*) gene, located near the *TRMT6* gene. Liver samples carrying minor alleles of rs236110 C > A (CA and AA) have significantly lower expression levels of the *MCM8* gene than those with CC alleles (Figure 1), suggesting the potential impacts of the SNP on the expression of crucial genes.

## 4 DISCUSSION

Many keystone studies have shown that paediatric cancer is characterized by a low burden of somatic mutations but a relatively high frequency of germline variants.^24^, ^25^, ^26^, ^27^ Unlike adult cancers, the few recurrent somatic mutations are insufficient to interpret paediatric tumours' initiation and clinical heterogeneity and to facilitate precision therapies. Instead, several studies suggest that specific pathogenic germline variants are promising in directing clinical management and risk stratification for solid tumours in children.^28^, ^29^ Therefore, discovering disease‐predisposing germline variants in paediatric neoplasms is indispensable for clinical decision‐making, disease surveillance and risk evaluation for patients, parents and siblings.

This study aimed to interrogate whether genetic variants in the *TRMT6* gene predispose to hepatoblastoma. Our results demonstrated that three *TRMT6* polymorphisms (rs236170 A > G, rs236188 G > A and rs236110 C > A) were able to modify hepatoblastoma risk in Chinese children individually. Moreover, the four studied SNPs collaboratively affected susceptibility to hepatoblastoma. We previously reported several hepatoblastoma‐predisposing genes that regulate different types of RNA methylation, including *YTHDF1*,^17^ *YTHDC1*,^14^ *WTAP*,^12^ *WDR4* ^10^ and *METTL1*.^9^ For instance, the rs7766006 in the *WTAP* gene, encoding a ‘writer’ that facilitates N6‐methyladenosine (m^6^A) methylation of RNAs, decreased the risk of hepatoblastoma.^12^ YTHDF1 can recognize the m^6^A modification in the RNAs and regulate their stability. The *YTHDF1* rs6090311 G allele protects carriers from developing hepatoblastoma risk,^17^ and eQTL analysis elucidated the correlation between the *YTHDF1* rs6090311 polymorphism and downregulated expression of its surrounding genes.^17^

TRMT6 interacts with TRMT61A and assists the latter in installing m^1^A modification in RNA by receiving and binding to target tRNA. Elevated expression levels of TRMT6 have been observed in several cancers and often predict inferior prognosis.^1^, ^3^, ^4^, ^5^ Wang et al. reported that the knockdown of TRMT6 impaired glioma cells' proliferation, migration and invasion, as revealed by CCK8, colony formation, Edu, transwell and wound healing assays.^3^ Wang and colleagues demonstrated that TRMT6/TRMT61A accelerated liver tumorigenesis by mediating m^1^A methylation of PPARδ translation‐related tRNAs.^5^ The increased PPARδ protein products upregulated cholesterol synthesis, further stimulating hedgehog signalling and fueling liver CSCs' self‐renewal.^5^ Interestingly, the TRMT6/61A complex also assists the base methylation of tRNA‐derived fragments. Su et al. found abundant TRMT6/61A‐dependent m1A in 22‐nucleotides long 3′ tRNA fragments. TRMT6/61A‐mediated higher m^1^A modification ablated gene silencing functions of tRF‐3 s, consequently inducing unfolded protein response to maintain bladder cancer cells to survive stressful tumour microenvironment.^1^ Moreover, TRMT6 also promoted HCC progression via the PI3K/AKT signalling pathway.^4^ These studies indicate that TRMT6 is closely implicated in cancer. Ali et al. unveiled associations between genetic alterations and the degree of mitochondrial RNA modification, verified across various tissue types.^30^ They reported that *MRPP3* rs11156876 was significantly associated with increased methylation level of tRNA P9.^30^ *TRMT61B* rs11684695 TT genotype displays the highest methylation levels of RNR2 RNA among GG, TG and TT genotypes.^30^ They also demonstrated that genetic variants associated with altered RNA modification levels were disease‐causing among several disorders, such as abnormal blood pressure, breast cancer and psoriasis.^30^ Therefore, it is biologically reasonable that potential functional SNPs that affect the expression and function of TRMT6 may influence disease susceptibility.

Despite the exciting findings of the study, limitations are unavoidable. First, this study enrolled only participants of Han Chinese ethnicity. Therefore, our results may not be directly extrapolated to different ethnic groups. Second, the sample size was relatively moderate, especially the number of cases. Third, we did not explore the effects of these SNPs on clinical outcomes because we failed to obtain relevant information. Finally, function analyses should be conducted for the gene and significant SNPs.

In conclusion, we identified three hepatoblastoma susceptibility SNPs of the *TRMT6* gene. These findings may facilitate the development of screening tests in the context of genetic counselling and promote our understanding of genetic variants' contribution to hepatoblastoma susceptibility.

## AUTHOR CONTRIBUTIONS

**Lin Ma:** Investigation (equal); resources (equal); writing – original draft (equal). **Jinhong Zhu:** Investigation (equal); writing – original draft (equal); writing – review and editing (equal). **Jiao Zhang:** Investigation (equal); resources (equal); writing – original draft (equal). **Wenli Zhang:** Formal analysis (equal); investigation (equal); writing – review and editing (equal). **Yong Li:** Investigation (equal); resources (equal). **Zhonghua Yang:** Investigation (equal); resources (equal). **Suhong Li:** Investigation (equal); resources (equal). **Jiwen Cheng:** Investigation (equal); resources (equal). **Li Li:** Investigation (equal); resources (equal). **Jing He:** Conceptualization (equal); formal analysis (equal); funding acquisition (equal); investigation (equal); resources (equal); supervision (equal); writing – review and editing (equal). **Peng Liu:** Conceptualization (equal); funding acquisition (equal); investigation (equal); resources (equal); supervision (equal); writing – review and editing (equal).

## CONFLICT OF INTEREST STATEMENT

None declared.

## Supporting information


Table S1.

## Data Availability

All the data are available upon request from the corresponding authors.
